# Translation Arrest: A Key Player in Plant Antiviral Response

**DOI:** 10.3390/genes14061293

**Published:** 2023-06-19

**Authors:** Annemarie Vermeulen, Frank L. W. Takken, Victor A. Sánchez-Camargo

**Affiliations:** Molecular Plant Pathology, Swammerdam Institute for Life Sciences (SILS), University of Amsterdam, 1098 XH Amsterdam, The Netherlands; annemarie.vermeulen@student.uva.nl (A.V.); v.a.sanchezcamargo@uva.nl (V.A.S.-C.)

**Keywords:** antiviral resistance, NLR-mediated translational arrest, PTGS, symptom recovery, translational repression

## Abstract

Plants evolved several mechanisms to protect themselves against viruses. Besides recessive resistance, where compatible host factors required for viral proliferation are absent or incompatible, there are (at least) two types of inducible antiviral immunity: RNA silencing (RNAi) and immune responses mounted upon activation of nucleotide-binding domain leucine-rich repeat (NLR) receptors. RNAi is associated with viral symptom recovery through translational repression and transcript degradation following recognition of viral double-stranded RNA produced during infection. NLR-mediated immunity is induced upon (in)direct recognition of a viral protein by an NLR receptor, triggering either a hypersensitive response (HR) or an extreme resistance response (ER). During ER, host cell death is not apparent, and it has been proposed that this resistance is mediated by a translational arrest (TA) of viral transcripts. Recent research indicates that translational repression plays a crucial role in plant antiviral resistance. This paper reviews current knowledge on viral translational repression during viral recovery and NLR-mediated immunity. Our findings are summarized in a model detailing the pathways and processes leading to translational arrest of plant viruses. This model can serve as a framework to formulate hypotheses on how TA halts viral replication, inspiring new leads for the development of antiviral resistance in crops.

## 1. Introduction

With an ever-growing population, there is an increasing need to optimize crop production. Plant viruses alone account for around 47% of the emerging plant diseases in crops [1]. Viral transmission between plants often involves human handling, animal feeding, infected seeds, or vectors. Common vectors include insects, such as thrips, aphids, leafhoppers, and white flies. Combatting insect vectors with insecticides is often inefficient due to strong selection towards resistant individuals, rendering most insecticides ineffective within a few generations. Moreover, insecticides are often detrimental to beneficial insects, such as pollinators [2]. Since insecticide resistance increases in agricultural areas and viral spread is hard to control, enhancing the natural resistance of crop species is crucial to combatting viruses.

Viruses are obligate parasites that rely on host translational machinery for protein translation of viral RNAs and for viral replication. Therefore, plants can display passive resistance to a certain virus when the translation machinery of the plant species is incompatible with the viral replication cycle [3]. For instance, mutation or reduced expression of the genes encoding the eukaryotic translation initiation factors eIF4G and eIF4E or isoforms of these proteins can provide recessive resistance to certain viruses in a broad range of plant species [4,5,6]. Durable recessive resistance against viruses can indeed be provided by changes in genes encoding components of the translation machinery [7], highlighting the importance of understanding the mechanisms by which plant viruses hijack host translation. Besides passive resistance, plants possess at least two known types of active immunity against plant viruses: RNA silencing (RNAi) and immune responses mounted upon recognition of viral proteins by specific nucleotide-binding leucine-rich repeat (NLR) immune receptors. The RNAi pathway is highly conserved among eukaryotes and has been shown to control gene expression during plant development and in biotic and abiotic stress responses [8]. In plants, antiviral resistance mediated by RNAi leads to either post-transcriptional gene silencing (PTGS) to control infection by RNA viruses or transcriptional gene silencing (TGS) and PTGS to control infection by DNA viruses. RNAi is considered the primary antiviral defense, and many (plant) viruses have evolved viral RNA silencing suppressors (VSRs) to counteract the RNAi pathway [9,10,11,12,13,14,15]. A second layer of active defense against plant viruses is mediated by immune receptors of the NLR type. Unlike RNAi, which is triggered upon recognition of double-stranded viral RNA molecules, NLR-mediated resistance is activated upon recognition of a viral effector protein. Viral effectors can be, e.g., the coat protein (CP) or movement protein (MP) [16]. Typically, NLR-type immune receptors consist of either a coiled-coil (CC) or a Toll Interleukin 1 Receptor (TIR) domain (referred to as CNL and TNL, respectively), a nucleotide-binding (NB) domain, two ARC domains, and a leucine-rich repeat (LRR) domain. NLRs can be localized at the plasma membrane, in the cytoplasm, and/or in the nucleus of plant cells [16]. They recognize effectors either directly, through interaction mediated by the LRR domain, or indirectly, through a binding partner (the guard–decoy model) or via modification of a host protein (bait) [17,18]. Upon recognition, NLRs adopt an active state by exchanging bound ADP for ATP [19]. Activation often results in local cell death called the hypersensitive response (HR). However, the activation of certain NLRs by their cognate viral pathogen can also lead to extreme resistance (ER) in which cell death is not apparent [20]. Whether NLR activation induces HR or ER is not solely determined by the NLR protein, as the expression of NLRs in different genetic backgrounds or the overexpression of their eliciting effectors can turn an ER output into an HR [20,21,22]. It has been hypothesized that HR and ER are either the result of separate sequentially activated pathways or phenotypic variants of the same pathway [23]. ER is thought to confer resistance to viruses through a translational arrest (TA) mechanism, possibly in concert with abscisic acid (ABA) signaling, preventing viral spread from infected tissues through callose depositions at plasmodesmata [24,25,26,27,28,29,30]. Two well-studied NLRs that trigger a viral TA are potato (*Solanum tuberosum*) Rx1 and tobacco (*Nicotiana tabacum*) N, conferring resistance to potato virus X (PVX) and tobacco mosaic virus (TMV), respectively [25,28,31,32,33]. Both the CNL Rx1 and the TNL N induce viral TA, which suggests that TA is a shared output downstream of NLR activation during antiviral response [28].

Translational repression is a common process in plant cells that occurs during development or in response to stresses, such as UV irradiation, high boron concentrations, and virus infection [25,28,34,35,36,37]. It is proposed that TA has an advantage over RNA degradation because of its potential reversibility, allowing the re-entry of (host) transcripts into translation once stress conditions fade [38,39]. In antiviral immunity, repression of viral translation is triggered through multiple distinct processes, including PTGS, NLR-mediated immunity, and signaling downstream of the conserved leucine-rich repeat receptor-like kinase (LRR-RLK) NUCLEAR SHUTTLE PROTEIN (NSP)-INTERACTING KINASE 1 (NIK1) [40]. In NIK1-mediated resistance, virus-derived small RNAs (vsRNAs) trigger a global TA rather than a specific TA in response to infection with geminiviruses [40,41]. Viral recognition leads to TA in many ways, either virus-specific or broad-spectrum. This suggests that translational inhibition is an important factor for viral resistance in plants [25,28,39,42,43,44], yet research in this area remains limited, especially for NLR-mediated TA. This review aims to provide a comparison of PTGS and NLR-mediated TA of RNA viruses to describe the overlaps and differences between these antiviral defense mechanisms.

## 2. Translation of Viral Transcripts in Plants

Viruses are obligate intracellular parasites that rely on host factors for propagation. This section examines transcript adaptation and the translation strategies and optimization strategies that RNA viruses use to ensure viral replication.

### 2.1. Adaptations of Viral RNAs for Enhanced Translation

Viral RNAs must disguise themselves to mimic eukaryotic mRNA, evade the host immune system, and hijack the host machinery for translation. Eukaryotic mRNA has a unique 5′ cap structure essential for translation initiation. The cap structure, which consists of a methylated guanosine residue, binds translation factors and ribosomes to initiate protein synthesis. Additionally, eukaryotic mRNA has a 3′ poly(A) tail that protects the mRNA from degradation by exonucleases [45]. Many plant RNA viruses lack either a 5′ cap, a 3′ poly(A) tail, or both, preventing recognition and translation by the host machinery. Moreover, many plant RNA viruses are multicistronic, containing several open reading frames (ORFs) within the same RNA strand or encoding a polypeptide that is processed by proteases into multiple peptides. To overcome the lack of a 5′ cap or a 3′ poly(A) tail, viral RNAs have evolved adaptations to recruit ribosomes. These adaptations include, e.g., 5′ internal ribosome entry sites (IRESs), 5′ viral genome-linked protein (VPg), 3′ cap-independent translation enhancers (CITEs), and cap snatching [46]. IRES elements are highly structured RNA sequences that allow ribosomes to initiate translation from a position internal to the RNA molecule, bypassing the requirement of a 5′ cap structure. VPg is a protein covalently linked to the 5′ end of some viral RNAs, and it is thought to enhance translation initiation [46]. CITEs are RNA elements located at the 3′ end of some viral RNAs that enhance translation initiation by interacting with translation initiation factors. Interestingly, both 5′ IRES and 3′ CITE elements are also present in some eukaryotic mRNAs and are thought to stimulate translation during stress and developmental circumstances when cap recognition is hindered [47]. These elements consist of *cis*-acting secondary structures that recruit host initiation factors or ribosomal subunits, enabling protein synthesis [48,49,50]. Another strategy involves VPg, which plays a crucial role in the translation of viral RNA by interacting with host translation factors, such as eIF4E and its isoform eIF4isoE. VPg competes with the cap structure of host mRNAs to bind these factors, thereby inhibiting host translation and redirecting the host machinery towards viral translation [51,52]. In contrast, some segmented negative-stranded RNA viruses have developed an alternative mechanism called cap snatching to initiate translation [53,54]. Cap snatching involves the cleavage of the 5′ end of host cell mRNAs, which is then fused with the viral RNA. As a result, the modified 5′ regions of viral RNA become identical to the endogenous mRNA and can undergo the regular translation process [53,54]. For a detailed list of the strategies employed by different plant virus families, see [46].

### 2.2. Viral Translation

Different RNA virus families have developed diverse mechanisms to achieve efficient translation. Depending on the location of the encoded peptide sequence in the viral RNA, commonly used strategies include cap-independent translation, synthesis of subgenomic RNAs (sgRNAs), cap snatching, and translational recoding [55,56,57]. Cap-independent translation occurs at the first 5′ localized ORF and is regulated by IRES and 3′ CITE structures in the viral RNA. These structures interact with translation initiation factors, such as eIF4E and eIF4G, or directly with the ribosomal subunit through 18s rRNA, which stimulates host translation initiation complex assembly and translation of the viral RNA, mimicking cap-dependent translation of eukaryotic mRNA [48,49,50].

In positive-stranded RNA viruses, sgRNAs are synthesized from initial viral RNA by an RNA-dependent RNA polymerase (RdRp) encoded in the viral genome. RdRp recognizes subgenomic promoters in the viral RNA that give rise to different sgRNAs. These sgRNAs enable the host machinery to translate viral proteins located internally or at the 3′ end of the viral RNA [55]. Due to the dense coding of viral genomes, overlapping or adjacent ORFs often require translational recoding for translation. Translational recoding includes leaky ribosome scanning, non-AUG initiation, ribosomal codon read-through, ribosomal frameshifts, and translational bypassing. The mechanism used largely depends on the viral genus [58].

### 2.3. Optimization of Viral Translation

In addition to *cis* elements that regulate translation, viruses have evolved various strategies to achieve maximum translation efficiency. For example, a commonly observed phenomenon is the induction of a ‘host shut-off’ mechanism, where the translation of endogenous mRNA is suppressed [59]. This can be accomplished through interference with the cap-dependent translation of host mRNAs, resulting in a decrease in, among other things, host antiviral responses and an increase in viral RNA translation. Moreover, viruses have optimized translation by facilitating RNA cyclization, interfering with host translation initiation, and compartmentalizing translation in virus factories (VFs) [60,61]. Viral RNA cyclization is promoted through a specific type of 3′ CITE structure that binds to eIF4F, leading to the interaction of eIF4F with a hairpin structure at the 5′ end of the RNA. In turn, this results in the cyclization of translation [62,63]. This *cis*-element-stimulated cyclization has been demonstrated for viruses in the Tombusviridae family [62,63]. Multiple viruses interfere with host translation by targeting eIF4E, altering its phosphorylation status [64]. In plants, phosphorylated eIF4isoE shows an increased binding affinity for VPg and enhanced mRNA translation [65,66], perhaps favoring the translation of viral VPg-containing RNAs over host mRNA. VFs, viroplasms, inclusion bodies (IBs), or viral replication complexes (VRCs) are intracellular structures induced by viruses. These membrane-bound inclusion-like bodies or spherules concentrate viral RNA and proteins, potentially enhancing viral replication. The formation of VFs is often facilitated by viral proteins that manipulate membranes of the endoplasmic reticulum, mitochondria, peroxisomes, and/or chloroplast membranes [67,68,69,70,71]. Although VFs are primarily associated with RNA replication, they have also been suggested to play a role in viral translation and cell-to-cell movement in certain cases [72,73].

## 3. Immune Mechanisms Resulting in Translational Repression of Viral RNAs

**Figure 1:**
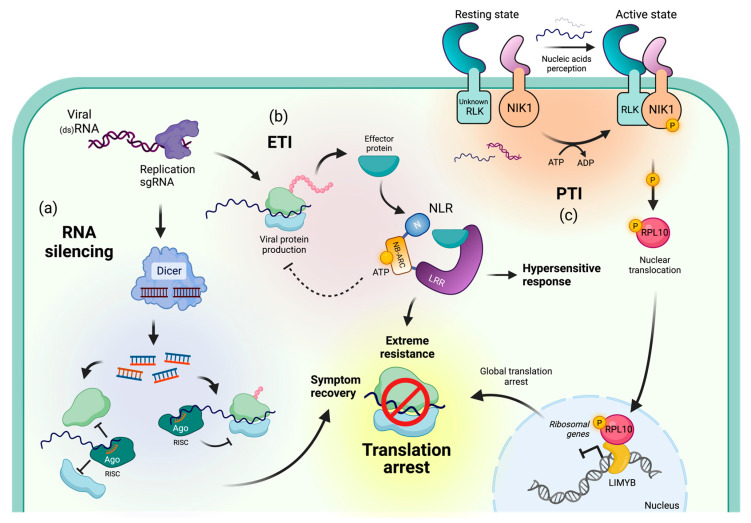
Distinct pathways trigger translation arrest in response to viruses. (**a**) Virus replication or subgenomic RNA (sgRNA) production for viral gene expression leads to the generation of double-stranded RNA (dsRNA). Proteins such as DICER that are part of the RNA silencing (RNAi) machinery recognize dsRNA, cleaving it into small RNAs (siRNAs) which are loaded into Argonaute (AGO) proteins, the catalytic subunits of the RNA-induced silencing complex (RISC). RISC selectively targets virus transcripts in a sequence-specific manner, leading to RNA cleavage or physical hindrance of ribosome assembly and progression, thereby causing translation arrest (TA). (**b**) Upon entry into the cell, viral components (proteins or nucleic acids) are recognized by intracellular receptors belonging to the nucleotide-binding leucine-rich receptors (NLR) family. This recognition initiates a signaling cascade that activates defense responses known as effector-triggered immunity (ETI). ETI is often accompanied by a hypersensitive response (HR) characterized by localized cell death at the infection site. Remarkably, ETI can also induce an extreme resistance (ER) response, which globally or specifically halts viral protein production irrespective of the presence of viral RNA, leading to the alleviation of symptoms. (**c**) Signals originating outside the cell can be perceived by receptor-like kinases (RLKs), which become activated upon binding to ligands, triggering pattern-triggered immunity (PTI). When PTI is induced via NIK1, NIK1 undergoes phosphorylation and phosphorylates downstream target ribosomal protein L10 (RPL10). Consequently, RPL10 is translocated to the nucleus, where it associates with the transcription factor LIMYB. The RPL10-LIMYB complex represses the expression of ribosomal genes, resulting in global translational repression. (Image created with Biorender.com).

Plants have developed diverse mechanisms to defend themselves against viral infections, including PTGS. PTGS, also known as RNAi, starts with the recognition of double-stranded RNA (dsRNA) that is often generated during viral replication [74]. Dicer-like proteins (DCLs) recognize and slice dsRNA into 21–24-nucleotide vsRNAs [74,75,76,77]. DCL4 is thought to be the primary DCL enzyme involved in the antiviral defense response against RNA viruses in plants, as it is required for the production of vsRNA and the cleavage of viral RNA molecules [78,79]. vsRNAs are loaded into Argonaute (AGO) proteins, which form the core of the RNA-induced silencing complex (RISC). RISC binds to target viral RNA molecules, leading to either cleavage and degradation or translational repression [80,81] (Figure 1a). Most RNA viruses encode VSRs to avoid PTGS. These VSRs either conceal vsRNAs to prevent RISC incorporation or impede proteins in the PTGS pathway by hindering their function, affecting their stability, or suppressing their expression [8,9,10,11,12,13,14,15].

### 3.1. Viral Recovery through PTGS-Mediated Translational Repression

Symptom recovery is observed in certain plant–virus interactions, where asymptotic leaves emerge or symptomatic leaves recover after systemic infection with a virus. This phenomenon has been reported for numerous unrelated viruses and can also be induced through mutation of VSRs [34,82,83,84,85]. Symptom recovery is associated with systemic sequence-specific resistance, providing protection against reinfections and cross-protection against related viruses [84]. Symptom recovery from RNA viruses can result from either degradation or translational repression of viral transcripts [42,43,86,87,88]. Symptom recovery is the only known phenomenon in plants where PTGS leads to translational repression [34,82].

In *Nicotiana benthamiana*, symptom recovery from tomato ringspot virus (ToRSV) is associated with PTGS but not accompanied by viral clearance [86]. Instead, ToRSV recovery is accompanied by an accumulation of the ToRSV RNA2 viral segment and a reduction in its encoded CP and MP through translational repression [42,87]. Whether translation of RNA1 of ToRSV is also repressed remains elusive. Similarly, symptomless recovery from an engineered tobacco rattle virus clone (TRV-GFP) infection in *Arabidopsis thaliana* is associated with a decrease in GFP fluorescence, followed by a drop in the viral titer [43,88]. During this stage, the TRV RNA is less associated with ribosomes, which results in reduced levels of GFP. This observation suggests that translational repression is involved in TRV recovery [43]. In contrast, in *N. benthamiana*, TRV RNA is known to be targeted for slicing [89]. In *A. thaliana* undergoing infection by oil-seed rape mosaic virus (ORMV), symptom recovery occurs in newly developed leaves in the presence of infection-competent replicating viral RNAs. It has been proposed that 21–22 nt siRNAs in concert with PTGS and TGS machinery are responsible for this recovery in a non-cell-autonomous manner, generating a source-to-sink siRNA gradient, where VSR function becomes saturated, perhaps inhibiting viral protein translation. Accordingly, *A. thaliana* mutants that result in a dysfunctional PTGS and/or TGS fail to undergo symptom recovery, while impairing RNA decay machinery enhances such a response, probably by allowing the accumulation of dsRNA, which can be processed into vsRNAs and exported to new leaves [90]. These data suggest that PTGS participates in translational repression during viral recovery. Moreover, under certain interactions, translational repression and slicing can occur in parallel [87].

### 3.2. NLR-Mediated Translational Arrest

During NLR-mediated TA, the translation of viral transcripts of the virus triggering the immune response is arrested, as well as that of transcripts from other viruses present in the cell [25,39,91] (Figure 1b). The resulting TA appears to be cell-autonomous and only occurs in cells where the NLR is activated [25,39]. Two structurally divergent NLRs, a CNL (Rx1) and a TNL (N), are known to induce TA in *N. benthamiana* [25,28]. How NLR activation leads to TA is poorly understood, and our current understanding of the Rx1- and N- pathway(s) leading to TA is discussed below. In contrast to NLR-mediated TA, LRR-RLK NIK1 triggers a global TA [40,41].

Rx1 has been introgressed into commercial potato cultivars from the wild potato species *Solanum andigena* [31,32,92]. The *Rx1* gene confers extreme resistance to PVX. In transgenic *N. benthamiana*, Rx1 is activated in the cytoplasm upon recognition of the PVX CP [32,93,94,95,96]. Recently, it was shown that Ran GTPase Activating Proteins 1/2 (RanGAP1/2) are targets of PVX CP [97]. Since inactive Rx1 and RanGAP2 interact through their CC and WPP domains, respectively, Rx1 activation likely occurs by indirect recognition through RanGAP2 [97,98,99]. Once activated, Rx1 is translocated to the nucleus, which is essential to the mounting of a full immune response [28,95,96,100]. Rx1 shuttling is mediated by RanGAP2 and the co-chaperone SUPRESSOR OF G2 ALLELE OF SKP1 (SGT1) [96,99,100]. In the nucleus, the activated form of Rx1 directly binds and distorts double-stranded DNA through its NB-ARC domain [93]. The DNA-binding capacity of Rx1 could enable it to function as a transcriptional regulator by facilitating access to DNA for transcription machinery. The binding specificity of Rx1 is suggested to be provided by the Golden2-like transcription factor (TF) *Nb*Glk1 [101]. In the absence of PVX, the CC domain of Rx1 binds to *Nb*Glk1 and a bromodomain (BD)-containing protein (*Nb*BDCP), preventing chromatin interaction [23]. Upon PVX infection, *Nb*Glk1 mediates Rx1 binding to Golden2-like consensus DNA sequences, which may lead to TA by regulating unknown target genes.

N mediates resistance to TMV through recognition of the p50 helicase domain present in the TMV replicase [102,103]. In *N. tabacum*, activation of this NLR results in HR [33]. However, when introduced in *N. benthamiana*, N activation leads to ER through TA [25,33]. Recognition of p50 is mediated by N-receptor-interacting protein 1 (NRIP1), which is normally localized in chloroplasts [104]. However, p50 recruits NRIP1 to the cytoplasm and nucleus to form p50-NRIP1 complexes [104]. Afterward, NRIP1 binds to the TIR domain of N, triggering activation [104,105]. The N-mediated defense response encompasses multiple pathways. However, the specific mechanism by which it induces TA remains unknown. Currently, the only protein suggested to be involved in TA is the helper NLR N REQUIREMENT GENE 1 (NRG1) via an unknown mechanism [39]. Future studies aimed at unveiling the proteins involved in NLR-mediated transcriptional activation could help determine whether both NLRs require the same partners for signaling or utilize distinct pathways. Differences in sensitivity to the VSR p38 of turnip crinkle virus (TCV) suggest that the mechanisms that lead to TA are different for N and Rx1 [28].

Alternatively, virus-derived nucleic acids from begomovirus, a DNA virus, can trigger a global TA through NIK1 [40,41] (Figure 1c). NIK1 is part of the same LRR-RLK subfamily as BRI1-ASSOCIATED RECEPTOR KINASE-1 (BAK1), which is well known for its role in plant defense against bacteria, fungi, and oomycetes [106,107,108]. The global TA following NIK1 activation is thought to be induced through the downregulation of translational-machinery-related genes [109]. This downregulation is indirectly facilitated by the ribosomal protein L10A (RPL10A), which translocates to the nucleus upon phosphorylation by NIK1, where it interacts with the L10-interacting MYB domain-containing (LIMYB) TF [41,41] (Figure 1c). However, the mechanism of NIK1 activation and the signaling pathway leading to global TA remain obscure, though transcriptional reprogramming might be common during TA in response to plant viruses.

## 4. Translational Repression

After recognition of a virus by the plant immune system, viral transcripts need to be recognized and their translation arrested. During TRV recovery and NLR-mediated TA, repression of viral transcripts is associated with an increase in RNA processing bodies (PBs) [39,43]. This section summarizes the current knowledge of how viral recovery and NLR-mediated TA impact transcript recognition, transcript halting, and PB formation. Furthermore, dependency on AGO proteins and VSR interference within these defense pathways will be discussed.

### 4.1. Transcript Recognition

To mount a TA of viral RNAs, the plant must distinguish viral RNAs from endogenous RNAs. Recognition of a viral transcript can occur through the sequence specificity brought by vsRNA in an RISC complex or by the binding of RNA-binding proteins (RBPs) to common viral motifs present in the RNA. Some of these common motifs include dsRNA, the 5′-ppp region of the RNA, the 5′ UTR region, and hairpin structures [110,111,112,113].

During viral recovery, transcript recognition is assumed to occur through sequence specificity provided by siRNAs [38]. This hypothesis is supported by the observation that plants have sequence-specific resistance to reinfection after viral recovery [84]. However, transcript recognition has not been reported for ToRSV or TRV recovery, where translational repression is involved. In NLR-mediated TA, viral transcripts present in the cell are halted [25,39,91]. Most likely, a common element present in viral RNA is recognized by an RBP, resulting in the translational repression of viral transcripts. Evidence suggests that a structural motif present in the CP-encoding part of the PVX RNA is essential for TA of these transcripts [25]. In correspondence with this, the translation of PVX transcripts lacking the CP region was no longer halted when N-mediated TA was triggered. The PVX-induced TA could be reverted when the CP ORF was replaced with another viral CP with low sequence similarity, suggesting that the recognition and subsequent targeting for repression is likely caused by a structural element in this region. Moreover, activation of the NLR Tm-2a from a wild relative of tomato (*Solanum lycopersicum*), *Solanum peruvianum,* inhibited the accumulation of PVX but not that of PVXΔCP in tobacco [25]. This suggests that the structural element present in the CP region might be a common target for NLRs. However, the possible binding partner and RNA structure both remain unidentified. Other elements of the viral RNAs, such as their 5′ cap structure and their poly-A tail at the 3′ region, are unlikely to be recognized during NLR-mediated TA, since TCV sgRNA lacks these structures and is still halted during N-mediated TA [39,114].

### 4.2. Translation Inhibition

After dsRNA sensors recognize the viral transcripts, the translation of transcripts can either be repressed before translation initiation or later, during translation elongation. For example, in miRNA-mediated translational repression, translation initiation is known to be inhibited through the physical blocking of mRNAs by AGO1-RISC binding at the 5′ UTR to prevent recruitment of the 48S ribosomal subunit and ribosome assembly [115]. In addition, depending on the position of the RISC target site, the binding of RISC to the RNA can physically block translation elongation [115]. The stage during translation at which the transcripts are halted can be determined through polysome profiling, as the association of ribosomes is prevented by translational inhibition prior to translation initiation [116]. During ToRSV recovery, monosome and polysome profiling revealed that translation inhibition occurred after translation initiation. However, during TRV recovery, translation inhibition appeared to occur before translation initiation [43,87]. In NLR-mediated TA, polysome profiles revealed that PVX RNAs were no longer associated with polysomes [39]. In N-mediated TA, PVX RNA was prohibited from associating with monosomes, which suggests that repression occurs before translation initiation [25]. However, the mechanism behind translation initiation inhibition during NLR-mediated TA remains obscure.

### 4.3. Processing Bodies

Liquid–liquid phase separation (LLPS) is a physical phenomenon where a homogeneous solution separates into two distinct liquid phases, driven by changes in concentration, temperature, or other factors [117,118]. RNA granules, which comprise all intracellular aggregations of ribonucleoprotein (RNP) complexes large enough to be microscopically visible, can undergo LLPS. Cells undergoing a viral infection often accumulate RNA granules, which dynamically influence each other by exchanging components, such as transcripts and proteins [119].

In plants, RNA granules can be classified into PBs, stress granules (SGs), and siRNA bodies. In PBs and SGs, LLPS behavior is thought to be driven by multivalent interactions between RBPs, RNA, and other proteins that enable the formation of weak reversible bonds, which, in turn, can dynamically change the properties of the granules [120,121,122,123]. PBs are essential in regulating gene expression, mRNA decay, and translation [124]. These bodies reside within cells to process aberrant mRNA transcripts. During mRNA decay, endogenous aberrant mRNA or viral RNAs are degraded through decapping by the mRNA decapping protein 2 (DCP2), followed by de-adenylation and 5′ to 3′ decay by the exoribonuclease XRN4 [124]. Decapping is assisted by the co-activator DCP1 and scaffold varicose (VCS) [125,126]. Besides mRNA decay, PBs are sites for storing translationally repressed RNAs [121]. Once the abundance of repressed RNA increases due to RNAi, virus infection, or UV irradiation, the number and size of PBs changes consequently [36,39,43,124,127]. Plant viruses have been proposed to utilize components of PBs for their benefit, and PBs have been suggested to store repressed viral RNA during immune responses and viral recovery [39,43,128,129].

During TRV recovery, *A. thaliana* shows an increase in PBs potentially containing repressed TRV RNA aggregates. Eventually, these RNAs are degraded by decapping enzymes present in these PBs. However, degradation of the TRV RNA is not necessary for recovery, as was demonstrated by the normal TRV recovery observed in the DCP2 mutant *its1* [43]. Although DCP1 was used as a PB marker, the exact composition of the PBs that form during TRV recovery remains unknown.

When cells underwent NLR-mediated TA, PBs were shown to accumulate as well [39]. These PBs contained DCP1 but were depleted of DCP2, resulting in transcript accumulation caused by the lack of RNA degradation [39]. The protein composition within these types of PBs remains unknown so far. After NLR activation, the cellular DCP1 levels remain unchanged, which suggests that a pre-existing pool of DCP1 acts as a nucleation point for PB formation [39]. While Meteignier et al. (2016) suggested that the lack of DCP2 in PBs could be due to limited levels of cellular DCP2, previous studies have shown that DCP1 and -2 are both recruited into PBs depending on the stress perceived and that this is not necessarily reflective of their cellular concentrations [130]. Therefore, it is likely that the depletion of DCP2 in NLR-mediated PB formation is an active exclusion rather than a passive reflection of PB protein component concentrations in the cytoplasm. However, the mechanisms that stimulate PB formation during NLR-mediated immunity remain to be elucidated.

The formation of PBs in response to NLR activation occurs through a different mechanism than that triggered by UV irradiation or RNAi [36,39]. For example, UV irradiation triggers phosphorylation of eIF2a, resulting in a global TA [131], which does not occur after N activation [39]. Additionally, when PTGS is repressed by VSR P19, PB formation is reduced, while P19 has no influence on PB formation following N activation [39]. Although the mechanisms that induce PB formation after NLR activation are distinct, whether the formed PBs are qualitatively similar or distinct remains under debate and requires further research. Whether PBs form during ToRSV recovery is unknown. However, it is unlikely, since the inhibition of translation initiation is known to result in PB formation, whilst inhibiting translation elongation leads to a decrease in PBs [36]. As discussed, translational repression during ToRSV recovery likely happens during elongation, whilst TRV recovery and NLR-mediated TA occurs before translation initiation. However, the exact mechanisms underlying PB formation have not been investigated for these viruses.

### 4.4. Translation Repression Depends on AGOs and VSR Interference

AGOs form an integral part of the RNAi pathway, where they act as the catalytic subunit of the RISC complex. Multiple AGOs (AGO1, AGO2, AGO4, and AGO7) are known to be involved in and are essential for antiviral immune responses [25,43,80,132,133,134,135,136]. All these AGO proteins have RNA slicing activity, whilst only AGO1 has been proven to be directly involved in translational repression [38,80,114,115,137,138]. AGOs can directly perform RNA slicing through their endonucleolytic PIWI domain [139]. Although the composition of the AGO protein interactome remains elusive, translational repression by AGO is thought to operate through association with WG/GW motif-containing proteins [140,141,142,143]. In *A. thaliana*, genetic studies identified a gene encoding the GW-containing protein SUO involved in miRNA-mediated translational repression [138]. However, whether SUO directly interacts with AGO through its GW motifs to perform its function has not been investigated, and how AGOs determine whether to perform slicing or repression is poorly understood. Possibly, AGO function is regulated through post-translational modifications, such as phosphorylation or changes in subcellular localization [42].

Multiple VSRs interact with AGOs to impair RNAi. For instance, AGO1 expression, stability, and activity can be targeted by VSRs [8,14,87,137,144,145]. In line with this, VSRs, including the carmovirus p38 and ipomovirus P1, contain the AGO-interacting motif WG/GW and compete with host proteins for AGO binding [144,146]. Some VSRs have multiple strategies to interfere with plant signaling. For example, p38 of TCV prevents the processing of dsRNA into siRNA and impairs siRNA loading into AGO1 and AGO2 in *A. thaliana* [144,147,148].

During symptom recovery of ToRSV in *N. benthamiana*, translational repression is AGO1-dependent [42]. It is likely that AGO1 is (in)directly involved in the PTGS mechanism leading to translational repression of ToRSV RNA2. This is supported by the finding that ToRSV CP hinders AGO1 function through interaction with the WG/GW motif of AGO1, triggering AGO1 degradation and probably competing with cellular WG/GW proteins involved in translational repression [13,87]. During TRV infection in *A. thaliana*, AGO2 and AGO4 are involved in initial susceptibility to TRV, whilst other unidentified proteins are required during recovery [43]. Multiple VSRs are known to affect symptom recovery and the expression of strong VSRs, such as HC-Pro from potyvirus, and p25 from PVX can eliminate symptom recovery during ToRSV infection [149]. Similarly, viral recovery of TRV was abolished by p38 of TCV [43]. Moreover, the inactivation of VSRs can lead to symptom recovery of virus isolates that typically do not display recovery. This has been shown for 2b of cucumovirus, HC-Pro of potyvirus, and P19 of tombusvirus [150,151,152,153]. Recovery in ToRSV and TRV is not dependent on a lack of VSRs in the virus isolates but rather on the relative strength of the VSRs present. As mentioned earlier, ToRSV CP acts as an AGO-hook triggering AGO1 degradation, and TRV 16K acts as a VSR by preventing AGO4-RISC assembly [87,154]. Additionally, the loss-of-function mutants for AGO1 and other genes involved in siRNA signal amplification, such as *RDR6*, *SGS3*, and *DCL4*, fail to alleviate infection symptoms [90]. These observations together suggest that AGO protein(s), in complex with partners containing WG/GW motifs, could participate in the TA of RNA viruses and that VSRs target this function.

Rx1-mediated and N-mediated TA are AGO1-independent and AGO4-dependent [25]. AGO4 is involved in resistance to many viruses through the RNA-directed DNA methylation (RdDM) pathway, yet it is also involved in antiviral mechanisms unrelated to RdDM in the cytoplasm [135,155,156]. During plantago asiatica mosaic virus (PIAMV) infection, AGO4 is suggested to re-localize to the cytoplasm and directly target the PIAMV RNA [157]. The exact mechanisms behind this interaction remain unknown. It is likely that AGO4 acts independently of the RdDM pathway as well during NLR-mediated TA, since the viral replication cycle is restricted to the cytoplasm. Involvement of the AGO proteins in NLR-mediated TA is further proven by the expression of VSR p0 from beet western yellows virus (BWYV), p38 of TCV, and p19 of cymbidium ringspot virus (CymRSV) [25,28,158]. p0 inhibits N-mediated TA and is known to induce the degradation of AGO proteins in *A. thaliana* [25,146]. However, its effect on Rx1-mediated TA has not been tested. Possibly, N-mediated TA is repressed by p0 promoting the degradation of AGO4. Besides p0, p38 of TCV inhibits N-mediated TA [25]. However, p38 does not physically interact with *At*AGO4, suggesting that N-mediated TA requires additional AGO proteins or that p38 inhibits TA at the dsRNA processing level [144]. The latter is unlikely since NLR-mediated transcript recognition is supposed to occur through common motif recognition rather than through siRNA complementarity, suggesting that the process might be siRNA-independent. Rx1-mediated TA is not inhibited by p38, suggesting a distinct mechanism leading to Rx1-mediated TA [28]. p19 of CymRSV is known to repress systemic PTGS by binding dsRNAs in order to prevent incorporation into RISC complexes. However, TA induced by both Rx1 and N is not influenced by p19, further supporting a distinction between the TA mechanisms involved in NLR and viral recovery [28,133,158].

## 5. Discussion

### 5.1. Similarities between TRV Recovery and N-Mediated Translational Repression

Although multiple differences exist between the mechanisms of viral recovery and NLR-mediated TA, there is potential overlap in the pathways controlling TRV recovery and N-mediated TA, as both are inhibited by the VSR of TCV, p38, and both induce the accumulation of PBs [25,39,43]. Since p38 can inhibit TA at multiple points, both through its function as an AGO hook and as an inhibitor of siRNA processing, it is impossible to elucidate whether the pathways overlap just because they are inhibited by p38 [144,147,148]. Further research is needed to uncover potential overlap in these mechanisms, including the testing and comparison of responses to other viral VSRs and dependence on other AGO proteins. Moreover, the composition of PBs formed during TRV recovery and NLR-mediated TA is mostly unknown, and caution should be taken when comparing these systems, since TA during TRV recovery has been studied using TRV-GFP, which may occur through a different route than recovery for wildtype TRV [43]. Furthermore, it remains unclear whether PBs during TRV recovery are depleted of DCP2, similar to the PBs formed during NLR-mediated immune responses. Additional research is needed to determine the exact RNA and protein contents of PBs and the mechanisms behind their formation.

### 5.2. Processing Bodies

The components present in the PBs formed during TRV recovery and NLR-mediated TA are mostly unknown. However, many proteins found in *A. thaliana* PBs where mRNA decay occurs have implied roles in translational repression, including DCP5, VCS, and SUO [38,126,138,159]. Therefore, these proteins might be present together with DCP1 [39,43]. Although it seems likely that PBs consist of viral RNA during viral recovery and NLR-mediated TA, their exact RNA content has yet to be determined. Furthermore, the exact mechanisms behind PB formation remain unknown, although recent studies suggest that DCP5 and RNA helicase proteins are required for PB formation in *A. thaliana* [160,161]. Furthermore, tandem zinc finger 1 (*At*TZF1) is involved in RNA binding and protein recruitment into PBs [162,163,164]. However, *At*TFZ1 appears to be mainly localized in root tips and the vasculature, and therefore it is unlikely that *At*TFZ is involved in PB formation observed during recovery and NLR-mediated TA, though other TFZ homologs might provide a similar function. In animals and yeast, granule formation is stimulated by peptide motifs within intrinsically disordered regions that are often found in proteins that reside in RNP complexes [165,166,167,168]. Whether plant PBs are driven by protein aggregation based on disordered regions has not been investigated in detail [169]. In animal cells, the proteome content of RNA granules was revealed using proximity labeling [170,171,172]. The use of this technique could aid in uncovering the protein content of plant PBs. However, purifying liquid membrane-free organelles remains challenging, and the exact content inside plant PBs remains unknown.

### 5.3. Gene Regulation during NLR-Mediated TA

Many questions remain unanswered regarding NLR-mediated TA, particularly whether transcription is necessary for TA and, if so, which genes are regulated. In Rx1-mediated TA, Rx1 may be directly involved in gene regulation by binding to Golden2-like *cis*-regulatory promoter elements via *Nb*Glk1 [97,101]. Chromatin immunoprecipitation followed by high-throughput sequencing (ChIP-seq) experiments could help identify which genes are regulated during the defense response, including those involved in NLR-mediated TA. The mechanism by which N-mediated TA is induced is also still unclear—whether it occurs through the degradation of a negative regulator or transcriptional reprogramming by WRKYs, SPL6, or another unidentified TF [173,174,175]. Conducting transcriptomic analysis of TMV infection on *N. benthamiana* carrying N could identify candidate genes involved in defense responses. Time-resolved RNA-seq could be used to reveal gene expression patterns and identify regulators involved in the N-mediated immune response.

### 5.4. Transcript Recognition during NLR-Mediated TA

The recognition of transcripts during NLR-mediated TA is likely provided by the structure of the CP coding region of the viral RNA. However, the protein(s) responsible for the recognition of this structure is unknown [25]. One possibility is that AGO4 is involved in transcript recognition independently of vsRNA complementarity. In human HeLa cells, multiple AGO isoforms, tethered to RNA as fusion proteins, were shown to repress translation based on hairpin structures present in the 3′ UTR of a reporter mRNA. This indicates that these AGOs can indirectly perform translational repression independently of small RNA templates [176]. It is plausible that plant AGO4 is able to recognize common structural motifs in viral RNAs via an unknown bound co-factor during NLR-mediated immunity, leading to TA.

The connection between the siRNA pathway and NLR-mediated plant immunity is not well understood. However, a recent preprint by Nielsen et al. (2023) proposes that DCL proteins are the main sensors of dsRNA in plants. Specifically, DCL4 has been identified as a major player in the antiviral siRNA response, while DCL2 is considered partially redundant. *A. thaliana* null mutants in *dcl4* exhibit impaired growth and show enrichment of WRKY DNA-response elements in the promoters of upregulated genes [177]. WRKYs are well-known TFs involved in plant defense responses against various pathogens [178]. Notably, some *dcl4* mutant plants exhibit an increased level of cell death. However, when the *dcl4* mutant background is combined with mutations in the co-chaperones *sgt1b* and *hsp90* (*heat shock protein 90*), both implicated in NLR decay, the penetrance of the cell death phenotype is increased, suggesting a connection between the siRNA pathway and NLR-mediated immunity [177]. Moreover, the loss of function of two non-related genes encoding NLR proteins in *A. thaliana*, the CNL L5 and the TNL RPP9/RAC1 (Resistance to *Albugo candida* 1), were found to suppress the *dcl4* autoimmune phenotype, establishing crosstalk between RNAi and NLR pathways for the first time. The capacity of both plant defense systems to communicate could explain the efficiency of the TA induced by RISC and NLR proteins, which, when combined, could account for a highly efficient antiviral response. Further studies are needed to unravel the molecular mechanisms underlying this crosstalk.

In animals, RNA sensing is primarily performed by immune receptors themselves. Some RIG-I-like receptors (RLRs) recognize dsRNA structures in viral RNA through intrinsic DExD/H-box RNA helicase domains [179,180]. In contrast, other RLRs and nucleotide oligomerization domain (NOD)-like receptors (NLRs) form complexes with RNA helicases that serve as co-factors for RNA sensing [181,182]. Moreover, in animal cells, stress granules induced during viral infection contain RLR immune receptors [183,184]. In plants, Rx1 has been shown to have the ability to bind single-stranded RNA [93], indicating that plant NLRs themselves could recognize transcripts in TA and that possibly they could also be found in PBs.

### 5.5. Translation Inhibition Hypothesis

**Figure 2:**
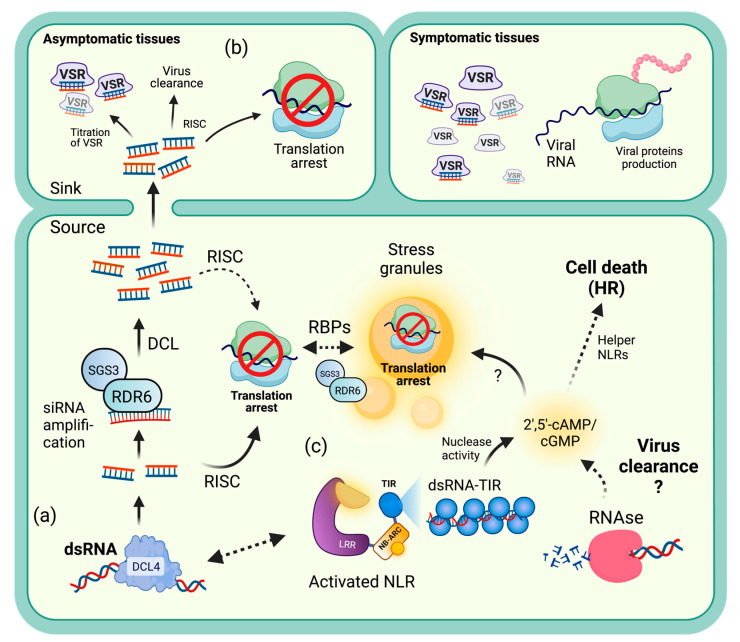
Crosstalk between the RNA silencing pathway and NLR-mediated translation arrest (TA) during antiviral immunity. (**a**) Upon detection of virus-derived dsRNA by DCL4, the RNAi pathway is activated. RNA-dependent RNA polymerase 6 (RDR6), in complex with Suppressor of Gene Silencing 3 (SGS3), can amplify the generated siRNA molecules. (**b**) Amplified siRNAs can be transported from infected leaves to new tissues, saturating viral silencing suppressors (VSRs) and enabling RISC to induce TA, resulting in asymptomatic leaves. (**c**) Active NLRs containing TIR domains (TNLs) and TIR-only proteins can bind and degrade RNA in a sequence-independent manner. Both RNAases and TIR-nuclease activity produce 2′,5′-cAMP, which is able to stimulate the formation of stress granules—membrane-free organelles—where TA occurs. SGS3-RDR6, along with other unknown RNA-binding proteins (RBPs), are known to induce stress granule formation in response to stress. RNA granule formation is a common outcome following immune receptor activation and cytosolic dsRNA perception through the RNA silencing pathway. Thus, the TA associated with RNA granules is proposed as one of the possible mechanisms for extreme resistance upon immune activation. (Image created with Biorender.com).

Replicating or transcribing RNA viruses produce dsRNA intermediates that could be perceived by cytoplasmic sensors such as the DCL proteins, triggering a response similar to pattern-triggered immunity (PTI), activating the siRNA pathway (Figure 2a). Amplification of this vsRNA signal could lead to saturation of VSR activity in a non-cell-autonomous manner, leading to new asymptomatic leaves, regardless of the presence of functional viral genomic RNA particles. In new leaves, the function of AGO-containing RISC complexes could be the main factor responsible for TA [90] (Figure 2b). However, information regarding the involvement of additional RBPs remains elusive.

During ETI, NLRs are activated and can act in different ways. For example, CNLs such as Rx1 may recognize secondary structures on the PVX genome, specifically at the *CP* ORF, leading to TA [25]. Whether Rx1 directly binds PVX RNA or whether accessory proteins are needed remains unknown. A chimeric NLR where the CC-NB domain of Rx1 was replaced by the homologous region of potato Gpa2, an NLR responsible for the resistance to the nematode *Globodera pallida*, conferred ER to PVX, suggesting that this module works in a non-pathogen-specific manner, while changing the recognition specificity of LRR directs the resistance against taxonomically unrelated pathogens [185].

Recently, 2′,5′-cAMP was shown to accumulate during different types of abiotic stress [186]. Both accumulation and exogenous application of this cyclic nucleotide induced the formation of SGs—RNA bodies that are able to promote TA. More recently, proteins containing TIR domains, such as NLRs and TIR-only proteins, were demonstrated to produce 2′,3′-cAMP/cGMP via DNA and RNA hydrolysis upon activation [187] (Figure 2c). When 2′,3′-cAMP/cGMP synthetase activity is combined with the NADase activity of oligomerized TNLs, cell death is triggered by promoting the association of ENHANCED DISEASE SUSCEPTIBILITY 1 (EDS1)-SENESCENSE ASSOCIATED GENE 101 (SAG101) with NRG1, resembling an HR [187]. ER could be achieved by halting viral translation and/or viral RNA clearance, which can be facilitated by the siRNA pathway and PBs, and are dependent on AGO proteins. Whether the 2′,3′-cAMP/GMP synthetase activity of TIR, utilizing RNA as a substrate in the absence of NADase activity, could potentially confer extreme resistance and trigger TA via virus elimination and the promotion of SG assembly remains to be ascertained.

Another layer of protection against viral infection that could lead to TA occurs at the translation machinery level, including the usage of isoforms of translation factors, the degradation of critical proteins for virus translation, and post-translational modification [188,189,190,191]. In plants, specific ribosomal proteins (RPs) have been demonstrated to be necessary for the translation of RNA viruses [192]. Therefore, it is conceivable that plants can adjust their RP composition in response to viral infection. The function of subunits of the translation initiation complex, such as eIF4E and eIFiso4E, can be inhibited by phosphorylation mediated by SnRK1 (SUCROSE NONFERMENTING 1-RELATED KINASE 1), a kinase implicated in metabolic signaling, which triggers a global inhibition of translation [193]. Interestingly, plants that overexpress SnRK1 show enhanced resistance to viral infection, which means that there is a direct link between plant immunity and signaling from the primary metabolism. Likewise, the master regulator TOR kinase, under favorable conditions, promotes anabolism [194]. However, under nutrient deficiencies or certain types of abiotic stress, TOR is inhibited by SnRK1 and catabolic pathways are activated [195]. TOR is also known for its role in the transcriptional regulation of plant immunity [39,196,197]. When TOR is pharmacologically inhibited, plants exhibit a primed resistance against pathogens in concert with salicylic acid signaling [198]. Taken together, a working model of antiviral immunity in plants can be proposed that involves several signaling pathways, such as PTI, ETI, and siRNA. The cellular compartment where the processes occur could also define the output, including the nucleus, in cytosolic non-membranous organelles, such as processing bodies, and in the cytosol. Finally, the metabolic status of the plant not only shapes the immune response against biotic challenges but likely also affects the response to abiotic stress. This influence could be magnified with TA—a common element that apparently links the response to these different types of stress.

## 6. Conclusions and Perspective

This review highlights our current understanding of the resistance mechanisms that lead to viral translational repression. Although many pathways appear to be involved, they ultimately result in the same outcome: viral TA. While distinctions and similarities in the underlying mechanisms have been identified through the influence of VSRs, significant gaps in our understanding of the pathways involved in viral recovery and NLR-mediated TA still exist. To address these gaps, additional research is needed to unravel the pathways and processes that lead to translational repression. This includes efforts focusing on transcriptomics and the (RNA-binding) protein composition of processing bodies. Furthermore, recovery to distinct virus types and NLR-mediated TA should be studied to obtain a more comprehensive view of the pathways involved in viral RNA translational repression. The evolution of multiple pathways leading to viral translational repression has been beneficial for plants, as it created a robust immune system that is hard to target by a single VSR encoded in a viral genome. Still, it poses challenges for research in this area. Ultimately, a better understanding of the mechanisms leading to translational repression will be of great value for designing crops with broader and more durable resistance to biotic (and possibly abiotic) stresses.

## Data Availability

Not applicable.
